# Development and Implementation of MyPainHub, a Web-Based Resource for People With Musculoskeletal Conditions and Their Health Care Professionals: Mixed Methods Study

**DOI:** 10.2196/63780

**Published:** 2025-02-24

**Authors:** Kerrie Evans, Jonathan Ko, Dragana Ceprnja, Katherine Maka, Darren Beales, Michele Sterling, Kim L Bennell, Gwendolen Jull, Paul W Hodges, Marnee J McKay, Trudy J Rebbeck

**Affiliations:** 1 The University of Sydney Sydney Australia; 2 Healthia Limited Bowen Hills Australia; 3 Western Sydney Local Health District Westmead Australia; 4 Curtin University Perth Australia; 5 The University of Queensland Brisbane Australia; 6 The University of Melbourne Melbourne Australia

**Keywords:** clinical pathways, allied health, self-management, health information, ehealth, co-design

## Abstract

**Background:**

Musculoskeletal conditions, including low back pain (LBP), neck pain, and knee osteoarthritis, are the greatest contributors to years lived with disability worldwide. Resources aiming to aid both patients and health care professionals (HCPs) exist but are poorly implemented and adopted.

**Objective:**

We aimed to develop and implement MyPainHub, an evidence-based web-based resource designed to provide comprehensive, credible and accessible information for people with, and HCPs who manage, common musculoskeletal conditions.

**Methods:**

This mixed methods study adhered to the New South Wales Translational Research Framework and was evaluated against the Reach, Effectiveness, Adoption, Implementation, and Maintenance (RE-AIM) framework. Consultation with key stakeholders (patients, HCPs, researchers, industry, consumer groups, and website developers) informed content, design, features, and functionality. Development then aimed to meet the identified need for a “one-stop shop”—a central location for information about common musculoskeletal conditions tailored to a person’s condition and risk of poor outcomes. MyPainHub was then developed through an iterative process and implementation strategies were tailored to different health care settings. Quantitative and qualitative evaluation occurred with patients and HCPs.

**Results:**

In total, 127 stakeholders participated in the development phase; initial consultation with them led to embedding 2 validated screening tools (the Short Form Örebro Musculoskeletal Pain Screening Questionnaire and the Keele STarT MSK tool) in MyPainHub to guide information tailoring for patients based on risk of poor outcomes. Development occurred in parallel and feedback from stakeholders informed design and content including structure, functionality, and phrasing and images to use to emphasize key points. Consultation resulted in information for patients being categorized using key guideline-based messages (general information, your pathway, exercise, and imaging) while information for clinicians was categorized into assessment, management, and prognosis. Implementation occurred in different health care settings with the most effective strategies being interactive education via webinars and workshops. The evaluation phase involved web-based questionnaires (patients: n=44; HCPs: n=29) and focus groups (patients: n=6; HCPs: n=6). Patients and HCPs found MyPainHub user-friendly, acceptable, credible, and potentially able to support self-management. Patient participants identified areas for improvement such as including more specific information on preventative measures and pain relief options. Despite positive feedback, only 35% (10/29) of HCPs used MyPainHub with their patients. HCP participants identified challenges including insufficient training and lack of familiarity with using web-based resources in existing clinical workflows. Following implementation, the information contained on MyPainHub changed knowledge and practice for some patients and HCPs.

**Conclusions:**

Following extensive and iterative stakeholder engagement, MyPainHub was developed as an evidence-based web-based resource and perceived by patients and HCPs as user-friendly, credible, and acceptable. Active implementation strategies are required for adoption and implementation and greater training focusing on strategies to implement MyPainHub into clinical practice may be necessary.

**Trial Registration:**

Australian New Zealand Clinical Trials Registry ACTRN12619000871145; https://tinyurl.com/438kkyt3

## Introduction

Musculoskeletal conditions are characterized by pain and reduced physical function, often resulting in deterioration of mental well-being, increased susceptibility to developing other chronic health conditions, and loss of productive life years in the workforce [1]. Globally, musculoskeletal conditions affect 1.7 billion people and are ranked as the highest contributors to years lost to disease, above mental, respiratory, and cardiovascular diseases [1-3]. Among people presenting for care, low back pain (LBP), neck pain, whiplash associated disorders (WAD), and knee osteoarthritis are the most prevalent and disabling musculoskeletal conditions [1,4]. In Australia, the health care system operates as a mixed public-private model, with primary funding through Medicare, a universal public insurance system providing subsidized access to health care services, supplemented by private insurance options. The federal and state governments manage health care collaboratively, with federal oversight on Medicare and Pharmaceutical Benefits Scheme funding, while states are responsible for public hospital management and service delivery. Despite targeted government initiatives to mitigate musculoskeletal disease burden, such as the national strategic action plan for arthritis and funding under the chronic disease management plan, musculoskeletal conditions in Australia still account for 20% of the total burden of disease [5].

One factor contributing to this burden is poor implementation of guideline-based care [6,7]. Broadly speaking, clinical guidelines for LBP [8,9], neck pain [10,11], WAD [12], and knee osteoarthritis [13-16], contain similar recommendations for the provision of evidence-based care—screening for red flags and risk factors for poor outcome, providing patient-centered education (including options for care, advice to stay active), and avoiding unnecessary investigations and interventions. However, health care professionals (HCPs) often fail to screen for prognostic factors and continue to overuse nonrecommended treatments, imaging, pharmacological interventions, and surgery when managing musculoskeletal conditions [17-22]. Non–guideline-based care occurs across health care settings (ie, primary and tertiary care settings) despite evidence that these low-value practices do not improve health outcomes, waste health care resources and can be harmful [17,23-25].

Barriers to implementation and adoption of clinical guidelines include system-related factors (eg, funding models [26,27]) but also practitioner and patient-related factors. For example, lack of time, limited access to evidence-based resources, and discordant expectations are cited by both HCPs and patients as reasons why evidence-based recommendations are not followed [28-31]. In response to some of these challenges and the increasing trend for people to obtain health information from the internet, websites and other digital resources about musculoskeletal conditions have been developed [32]. The sheer volume of resources now available is overwhelming and information from the internet can be inaccurate, perceived to have low credibility, lack cultural diversity and not meet the needs of consumers [33-38]. In addition, often there is little training or education provided to HCPs as to how to best use internet-based resources to help implement, adopt and support evidence-based care [39-41].

To empower and engage patients and HCPs to make informed decisions about evidence-based recommendations, websites should be developed and co-designed by end users, include input from experts and opinion leaders, and be practical and scalable, that is, follow a translational research framework [42]. Examples of websites that have adopted a translational research framework include MyBackPain [33], MyWhiplashNavigator [43], and MyJointPain [44]. The development of these websites began with a needs assessment to identify research priorities and consumer needs before creating and curating guidelines and evidence-based health care information. Stakeholder engagement was essential in content creation and testing. A dissemination strategy was developed to ensure that the website was reached by the target audience. Monitoring and evaluation mechanisms tracked website usage and user feedback for quality improvement and their impact has been investigated in clinical trials [45,46]. Although these websites have undergone a robust and rigorous process of development, the information contained on these websites is condition specific. Given that many people experience multiple coexisting musculoskeletal conditions [47], and that clinical guidelines for common musculoskeletal conditions contain similar key messages [48], the aim of this study was to develop a website that could serve as a hub or a “one-stop shop” for both patients with, and HCPs managing, musculoskeletal conditions. The website was also initially developed to support patients and HCPs enrolled in a randomized control trial that was due to commence—the PAthway of CarE (PACE) musculoskeletal (MSK) clinical trial [49]. PACE-MSK, funded by Australia’s National Health and Medical Research Council (GNT1141377), was designed to evaluate the effectiveness of a clinical PACE for people with musculoskeletal disorders where care is provided based on people’s risk of poor outcome [49]. A website that could serve as a one-stop shop for both patients and clinicians in PACE-MSK, as well as support patients and HCPs beyond the trial, was an important part of the design. This study reports on the development, implementation, and evaluation of MyPainHub, a website designed to provide support to patients and HCPs in the management of LBP, neck pain, WAD, and knee osteoarthritis.

## Methods

### Overview

This mixed methods study involved 3 stages (development, implementation, and evaluation), and followed the New South Wales Translational Research Framework [42] with outcomes assessed according to the Reach, Effectiveness, Adoption, Implementation, and Maintenance (RE-AIM) framework [50]. Both frameworks provide structured approaches to ensure that research is relevant and scalable and are widely used for research in the Australian health care context.

### Ethical Considerations

Ethics approval was obtained from the Human Research Ethics Committees at The University of Sydney (2018/926, 2021/951), The University of Melbourne (1954239), The University of Queensland (2019000700), Curtin University (HRE2019-0263, HRE2020-0562) and Western Sydney Local Health District (2021/ETH11872). Participants were informed about the study before providing their written consent (either on paper or via a web-based form). Participation was voluntary and participants were informed about their right to withdraw from the study at any point without any consequences. Collected data were coded and deidentified.

### Recruitment

Each stage involved different recruitment procedures. During the development stage, researchers, consumer, industry and clinical partners were identified by members of the research team and invited to participate via direct contact. These stakeholders were those with recognized expertise in musculoskeletal conditions and who had previously collaborated with members of the research team or been involved in previous studies. HCPs were involved in the PACE-MSK trial or had been involved in previous trials and had consented to be contacted for future trials. HCPs were working in either primary or tertiary (hospital) settings. People with MSK conditions had been involved in previous trials and had consented to be contacted for future trials. Contact was made with these groups via email or direct phone call. Students allied to HCPs were enrolled in a unit of study at a participating university and had consented to participate.

In the implementation and evaluation phases, HCPs and patients were involved in the PACE-MSK trial or had been involved in previous trials and had consented to be contacted for future trials. In addition, HCPs working in occupational health settings known to the research team or who had been involved in previous studies were invited to participate either by email or direct phone call. Those HCPs working in our clinical partners’ tertiary care setting (Western Sydney Local Health District, [WSLHD]) were directly invited by their peers and managers. Patients with MSK conditions were also invited to participate by their treating clinician.

### Stage 1: Development

The aim of the development stage was to design a website where patients and HCPs could access relevant, up-to-date, high-quality evidence-based information on common musculoskeletal conditions (LBP, neck pain, WAD and knee osteoarthritis). Given the expected commencement date of the PACE-MSK trial, the development stage incorporated comprehensive stakeholder engagement rather than a formal co-design process. The problem identified by the research team was that multiple resources exist, with neither a central nor coordinated location for people to access credible evidence-based information [51].

The first step of the New South Wales Research Translation Framework is Idea Generation, where key stakeholders are engaged to design a solution to the problem. Our core team comprised clinician- researchers from 4 Australian Universities (The University of Sydney, The University of Queensland, The University of Melbourne and Curtin University) with research expertise in musculoskeletal conditions. The team determined that key stakeholders likely to benefit from this resource were allied HCPs working in both primary and tertiary care, researchers involved in creating evidence for musculoskeletal conditions, specialist musculoskeletal clinicians, specialist medical professionals, people with musculoskeletal disorders, consumer organizations and industry partners. We then determined the level of influence and interest of each stakeholder using a stakeholder engagement matrix and considered appropriate strategies for managing their involvement and expectations [52]. Engagement methods ranged from being involved in co-design and implementation (manage) to piloting of resources (meet needs) and meetings (keep informed; Table 1).

**Table 1:** Classification of key stakeholders involved in the development stage of MyPainHub according to influence and interest, appropriate strategies and engagement methods.

Key stakeholder	Influence and interest	Appropriate strategies	Engagement methods
Allied HCPs^a^, Specialist musculoskeletal allied HCPs	Higher influence, higher interest	Involve	Consultation on key content Facilitated meetings to review and refine content Focus groups to pilot and identify gaps Involvement in implementation process
Student allied HCPs	Lower influence, higher interest	Involve	Involve in implementation and evaluation process
Researchers involved in musculoskeletal health	Higher influence, higher interest	Involve	Consultation on key content and existing resources
Specialist medical professionals	Lower influence, higher interest	Meet needs	Semistructured interview on resources and pathway
People with musculoskeletal conditions	Lower influence, higher interest	Meet needs	Review and piloting of resources with feedback
Consumer partners: Arthritis Australia, Australian Pain Management Association, Musculoskeletal Australia	Higher influence, lower interest	Keep informed	In-person meetings
Industry and clinical partners: Australian Physiotherapy Association, Chiropractic Australia, State Insurance Regulatory Authority of New South Wales, Primary Healthcare Networks, Knowledge Translation Australia	Higher influence, lower interest	Keep informed	In-person meetings

^a^HCPs: health care professionals.

We held work-in-progress meetings with key stakeholders between July 2018 and October 2022. Discussions included design concepts, content, functionality, website maintenance and support and how this could be implemented. To ensure optimal functionality and ease of use, a digital agency (Vivo Group, Brisbane, Queensland, Australia) with experience in development of user-friendly websites was selected to design the website following a robust tendering process. As the content was developed, each stakeholder group shared their views and perspectives and provided feedback about additional key messages and resources that should be included or omitted. Before implementation, the website went through final checks by the research team and the digital agency to test features, functionality and resolve any issues.

### Stage 2: Implementation

The aim of the implementation stage was to determine strategies that would help ensure effective and scalable implementation of the website before its widespread public release. Groups thought likely to source information about musculoskeletal conditions were identified and included allied health students and graduate allied HCPs working in health care settings where people with musculoskeletal conditions present. Settings included primary care, occupational health, and tertiary care settings. Finally, allied HCPs working in a specialist capacity were engaged. This process ensured implementation occurred with clinicians with a range of clinical experiences and in different care settings. We chose implementation strategies ranging from passive (dissemination) to more active strategies (facilitated and discussion-based education with opinion leaders) based on strategies that had been successful in our previous work (Table 1) [33,43,44]. During this stage, the website was not publicly accessible.

Patients with musculoskeletal conditions were invited by their treating clinician to use the resources on the website and then provided consent to participate in qualitative studies associated with both our primary and tertiary care implementation initiatives. After using the website, patients participated in a semistructured interview (via a web platform) or focus group (in person), depending on what was feasible in the particular health care setting and on the patient’s preference and location, to provide their opinions on the resource.

#### Allied HCPs Working in Primary Health Care Settings

They were engaged via a passive (dissemination) strategy to use the website. The primary HCPs providing care for people with musculoskeletal conditions in the PACE-MSK trial [49] were informed of the trial and the website via a phone call. Both the HCP and their patients were emailed a link to the website and encouraged to refer to the resources during the episode of care. Over an 18-month period, the researchers held fourteen 1-hour video-conferenced noncompulsory educational meetings inviting HCPs to attend with the aim of keeping them engaged in the trial and website.

#### Allied HCPs Working in Occupational and Tertiary Care Settings

They were invited to participate in an active (interactive education using opinion leaders) implementation strategy. HCPs working in occupational health settings were invited to participate either by email or direct phone call. Those working in our clinical partners’ tertiary care setting (WSLHD) were directly invited by their peers and managers. Those accepting the invitation then attended a web-based (occupational health) or in-person (tertiary care) discussion-based educational workshop explaining the purpose of the website and its key features. These HCPs were then encouraged to use the resources on MyPainHub to assist in assessment and management of their patients. After completion of the episode of care (or after 3 months), the HCPs were invited to complete a questionnaire to evaluate acceptability, feasibility, credibility, and actual practice of using the website and then participated in a focus group to explore their opinions in more depth (Multimedia Appendix 1).

#### Specialist Allied HCPs

They were invited to participate in a 2-day facilitated discussion-based educational workshop described elsewhere [53]. The workshop was designed to inform specialist HCPs about how to implement risk-stratified care (supported by MyPainHub) for people at risk of poor outcome. Key features that may facilitate the provision of this care were discussed during the workshop, including how MyPainHub could be used to support management. Two follow-up web-based meetings were held to encourage use of the website and implementation of a new pathway of care.

#### Student HCPs

They were engaged through classroom education. The website was integrated as a learning resource in 2 units of study for physiotherapy students in their final year of study at The University of Sydney. Key components of the education model included (1) risk assessment using the web-based risk-assessment tools resources available on the website, (2) using the resources to assist the provision of evidence-based treatment (guideline-based exercises and risk-based advice) for people with musculoskeletal disorders, and (3) timely and appropriate referral to specialist musculoskeletal clinicians for people at high risk of poor outcomes.

#### Outcome Assessed

The main outcome assessed for the implementation stage was the reach of the strategy. Reach was primarily assessed by the number registered on the resource or those invited to use it. Website traffic and trends (eg, total visits, total page views, and top landing pages) were collected through Google Analytics and built-in website reports.

### Stage 3: Evaluation

The aim of the evaluation stage was to investigate both effectiveness, acceptability, and adoption of the website (RE-AIM framework) by allied HCPs (students, allied HCPs, and specialist HCPs) and people with musculoskeletal conditions. Mixed methods involving quantitative (web-based survey) and qualitative (focus groups or semistructured interviews) were used.

#### Allied HCPs (Students to Specialists)

Participants were invited to share their perspectives about MyPainHub via a web-based survey. Participants answered questions relating to change in knowledge and practice (eg, “The website has changed the way I use prognostic tools”), acceptability and credibility of the website (eg, “I trusted the information on the website”), structure and features of the website (eg, “It was easy to navigate through the website”), and overall satisfaction with the website. HCPs were also asked open-ended questions which explored opinions on the best features of the website, barriers to use and suggestions for improvement. Student HCPs were asked additional questions relating to whether the information on the website aligned with what they had been taught and helped them achieve the learning outcomes of their course (eg, “The messages on the website about imaging made sense to me”; “Viewing the resources in MyPainHub helped me achieve the learning outcomes of my course”).

Qualitative data were collected from a proportion of HCPs who had been directly invited to participate by their peers and managers and who had consented to participate in a focus group. Participants were provided with the option of completing only the web-based survey or participating in both the web-based survey and the focus group. The focus groups were conducted in person and were designed to explore HCPs’ opinions in greater depth.

#### People With Musculoskeletal Conditions

As occurred with HCPs, people with musculoskeletal conditions were invited to share their perspectives about MyPainHub via a web-based survey. Patient participants answered questions designed to assess (change in) knowledge (eg, “People with my condition should not exercise until they have had scans done”), acceptability and credibility of the website (eg, “I trusted the information on the website”), structure and features of the website (eg, “It was easy to navigate through the website”), and overall satisfaction with the website. Patient participants were also asked open-ended questions which explored opinions on the best features of the website, barriers to use and suggestions for improvement.

Qualitative data were collected from a proportion of people with musculoskeletal conditions who consented to participate in a focus group (in person) to explore their perspectives in greater depth. Participants in the PACE-MSK trial participated in semistructured interviews (via a web platform) 3 months after entering the trial but these findings will not be reported in this paper.

#### Data Analysis

With respect to the web-based surveys, closed-ended questions were rated using a 5-point Likert scale ranging from 1 (strongly disagree or extremely unsatisfied) to 5 (strongly agree or extremely satisfied). The open-ended questions underwent a descriptive analysis.

With respect to the qualitative data collected during the semistructured interviews and focus groups, the discussions were recorded and transcribed. Analysis was conducted first by familiarization, then preliminary coding, searching, reviewing and naming and then reporting [54]. The transcriptions were manually checked by a member of the research team and corrected against the verbatim account. The transcriptions were read several times to make sense of the data. Open coding was conducted by naming sections of the participant responses in the text. Codes were then grouped to form meaningful categories in discussion with all authors. The next step was to construct broad themes from the categories with authors working collaboratively in the interpretation of qualitative data to draw on the combined insights of all members of the research team.

## Results

### Overview

The total number of participants involved in each stage is provided in Table 2.

**Table 2:** Stakeholders involved in development, implementation and evaluation of MyPainHub.

Stages and stakeholders		Participants involved, n
**Development**		
	HCPs^a^	67
	Researchers	28
	Specialist medical professionals	6
	People with musculoskeletal conditions	18
	Consumer partners	3
	Industry and clinical partners	5
**Implementation**		
	HCPs	136
	People with musculoskeletal conditions	778
**Evaluation**		
	HCPs (web-based surveys)	29
	HCPs—focus group (tertiary care, in person)	6
	People with musculoskeletal conditions (web-based surveys)	11
	People with musculoskeletal conditions—focus groups (tertiary care, in person)	6
	People with musculoskeletal conditions—semistructured interviews (primary care, via a web platform)^b^	33

^a^HCPs: health care professionals.

^b^Findings not reported in the present study.

### Stage 1: Development

The stakeholder consultation generated ideas that informed the website design and implementation. This process informed the development of the website both for “Patients” and “Clinicians.” Key features and content were identified and then modified iteratively as a result of the consultation process. Modifications did not follow a strict, linear order occurring instead in parallel.

#### MyPainHub for Patients

The core clinician-researcher team initially identified that common key guideline-based messages for implementation among people with musculoskeletal conditions were to provide accurate advice, appropriate messaging regarding imaging, to promote exercise, and provide differential care based on risk of poor outcome. The stakeholder consultation process resulted in agreement to structure the website around prognostic risk and musculoskeletal condition, with resources automated thereafter.

Two risk tools, the Short Form Örebro Musculoskeletal Pain Screening Questionnaire (SF-ÖMPSQ) [55] and the Keele STarT MSK tool [56] were identified by the research team as being validated tools to risk-stratify people with musculoskeletal conditions. Initial surveys of the specialist HCPs (n=50) suggested that 44% (22/50) either frequently or always used the SF-ÖMPSQ, 14% (7/50) the WhipPredict tool [57,58], 8% (4/50) the Keele STarT MSK tool [56], and 6% (3/50) the STarT Back Tool [59,60]. However, given WhipPredict is specific for WAD and the STarT Back Tool is specific for LBP, it was decided to embed the SF-ÖMPSQ and the Keele STarT MSK tool (both designed for broader musculoskeletal conditions) on the website for patients to complete with scores being automatically calculated. After completing the tools and nominating their primary condition (LBP, neck pain, or knee osteoarthritis), patients access resources that are designed specifically for both their risk subgroup (according to their score on the SF-ÖMPSQ) and condition. Each time a patient logs into the website, they are taken to their condition and risk-specific home page but can access information for the other conditions through simple navigation links and drop-down menus where appropriate.

The stakeholder consultation process identified that people with musculoskeletal conditions (n=18) wanted general information about their condition, ideas on how they could adapt to their lifestyle, their work and what they could do to help themselves. Consultation with industry (Table 1) resulted in adding links to other evidence-based websites and resources (eg, the Royal Australian College of General Practitioner’s guidelines for the management of knee osteoarthritis) and highlighted that options for care should be clearly explained (eg, people at low risk of poor outcome should be encouraged to avoid imaging and surgery and be provided with evidence-based advice and education whereas those at high risk should be provided with more comprehensive education related to the multifactorial, biopsychosocial nature of pain disorders). Consultation with patients with musculoskeletal conditions resulted in information being written in a friendly and nonjudgemental way and links to other websites and resources were selected so that they were a “simple click away” to avoid users being sent to another website where they would have to create an account. The consultation process resulted in the resources being structured under the categories of “General Information,” “Your Pathway,” “Exercise,” and “Imaging.”

#### MyPainHub for HCPs

Initial stakeholder consultation with 67 HCPs resulted in >200 individual pieces of feedback about the content, structure, features, and resources to use. Feedback included, for example, modifications to phrasing used or emphasis of key points (eg, “More emphasis on ‘not one posture is best’ would be good and give permission to sit in a comfortable, relaxed position to reduce or prevent fear of movement”), additional information to add (eg, “You could add some info about basic exercise such as walking, range of movement, balance and light upper limb weights”), structure (eg, “It was very easy to navigate”), and appearance (eg, “I think the pages that have banner images should have those images reduced in size – the banners are little dominating”). This feedback, and changes made to the website, was iterative. Overall, the feedback suggested that the website should contain key guideline-based messages, include “bite-sized” videos of information delivered by credible HCPs, include key practice points, open-access contemporary articles, clinical guidelines and outcome measures, and links to other credible resources. Subsequently, the information on MyPainHub for clinicians was structured under the categories of “Assessment,” “Prognosis,” and “Management.”

#### Structure and Appearance

The website designers provided expertise on the color scheme, structure and functionality of the website to achieve the aims of the project. The content was written in a professional and friendly tone, and images were selected that avoided emphasizing, for example, a pathoanatomical focus where not appropriate and that avoided stereotyping.

**Figure 1:**
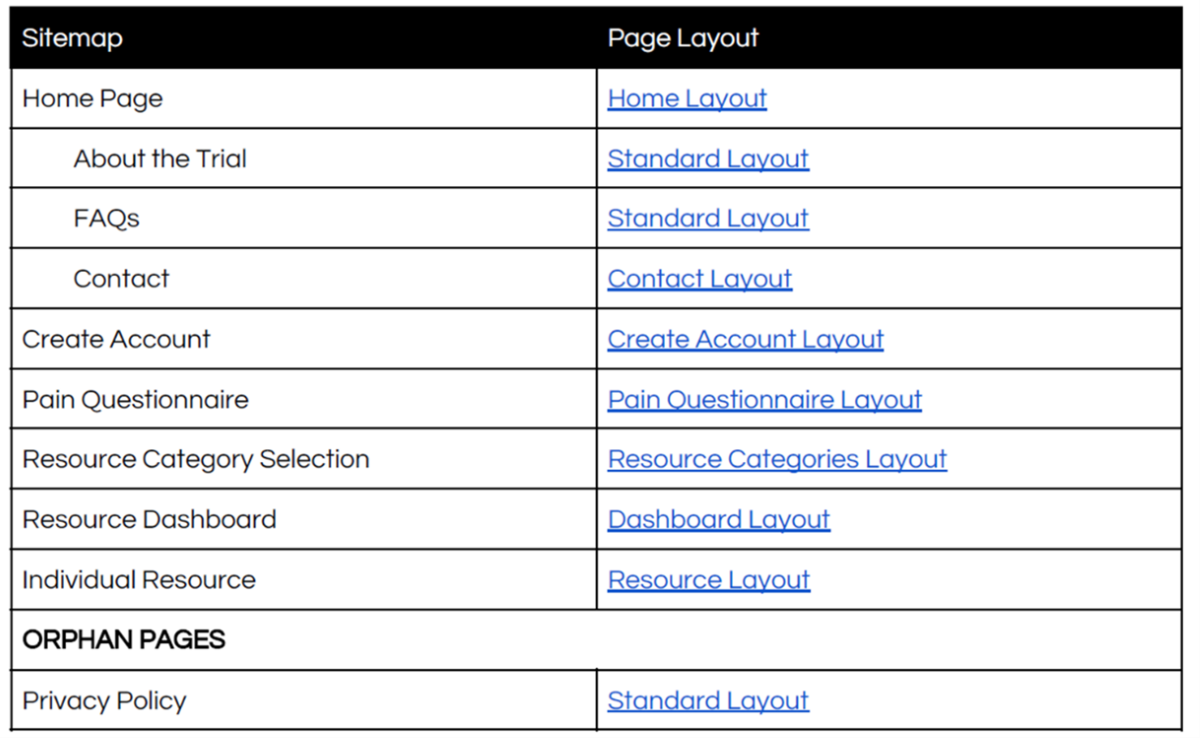
Sitemap for the development of MyPainHub.

**Figure 2:**
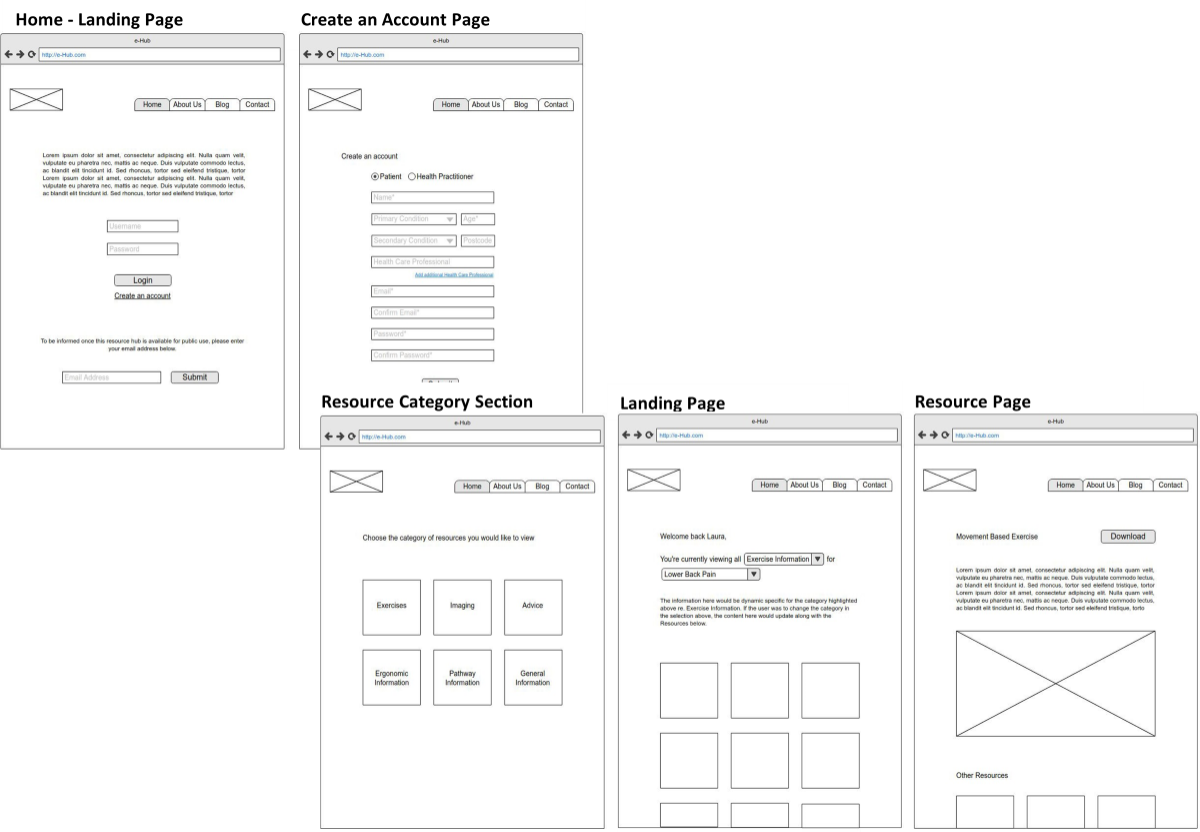
Wireframes used in the development of MyPainHub were designed to be clear and simple to facilitate navigation and clarity of information for the user.

The sitemap is shown in Figure 1. Pages were kept simple to optimize ease of navigation (Figure 2).

#### Permissions

The website was built on the website designer’s content management system which was developed in a software platform in PHP (using the Symfony framework) that is based on standard customizable modules. A tiered permission system was used for MyPainHub. Administrators (ie, researchers) were able to access and amend all information on the website. HCPs were able to review all content on the website. Patients were able to view content related to their risk profile (ie, low risk or high risk) but they could see information for all conditions. However, once they identified their primary complaint during their registration process, their landing page was set to their primary condition. Only the researchers had access to patients’ scores on the SF-ÖMPSQ and the Keele STarT MSK tool (during the PACE-MSK trial, the researchers would contact the patient’s treating HCP to inform them of their patient’s score [49]).

### Stage 2: Implementation

Implementation of the website took place in 4 Australian states (New South Wales, Queensland, Western Australia, and Victoria) from February 2018 to December 2023 as a component of PACE-MSK, the clinical trial for which the website was built [49]. As part of PACE-MSK, 767 people with musculoskeletal conditions had consented to be part of the trial and were therefore required to register on MyPainHub*.* A total of 693 HCPs were involved in the PACE-MSK trial but registering on MyPainHub, whilst possible, was not a requirement of being involved in the trial. In addition to the PACE-MSK trial, the website was implemented within WSLHD (both patients and HCPs), with allied HCPs in occupational health settings and with students (Table 3).

The implementation strategy adopted influenced the number of participants who, despite consenting to participate, registered on the website (Table 3). As a result of the education via webinars and workshops, 100% of allied HCPs working in occupational settings, tertiary care settings and specialist HCPs who consented to participate registered on the website. However, despite being embedded in a unit of study in a university physiotherapy program, only 16% of students registered on the site. Passive strategies, such as phone calls or reminders, were ineffective with only 29% of HCPs working in primary care registering (Table 3).

Website data for a 12-month period (June 2021-June 2022) are presented in Table 4. The total page views of the website during the period were 23025 of which 12149 were unique page views. The pages with the highest unique views were general information about knee osteoarthritis, neck pain assessment (clinicians), frequently asked questions, LBP assessment (clinicians), general information about neck pain (general information), and exercise for knee osteoarthritis (Table 4). Users viewed on average 4 pages during a visit and stayed approximately 4 minutes on each page.

**Table 3:** Target markets of website users, implementation strategies, the numbers invited in each target market and percentage successfully registered.

Target market	Implementation strategy	N=invited	N=consented, (participants)	N=registered, n (%)
HCPs^a^ in primary care	Reminders via phone calls and emails	693	196	57 (29)
Allied HCPs in occupational health settings	Discussion-based education (webinars) and reminders	48	6	6 (100)
Allied HCPs in tertiary care (WSLHD^b^)	Interactive education (in-person workshop) and reminders	9	6	6 (100)
Specialist allied HCPs	Interactive education (in-person workshop and 2 web-based workshop reminders)	128	50	50 (100)
Student HCPs	Embedded classroom education	102	102	17 (17)
People with musculoskeletal conditions in primary care	Invitation via treating HCPs and research team (PACE-MSK trial)	767	767	767 (100)
People with musculoskeletal conditions in tertiary care (WSLHD)	Invitation via treating HCPs	18	11	11 (100)

^a^HCPs: health care professionals.

^b^WSLHD: Western Sydney Local Health District.

**Table 4:** Summary of the number and duration of page views for MyPainHub.

Page title	Unique page views, N	Average time (seconds)
Knee osteoarthritis: general information	657	42
Neck pain: assessment (clinicians)	408	41
Frequently asked questions	157	107
Lower back pain: assessment (clinicians)	128	88
Neck pain: general information	121	47
Knee osteoarthritis: exercise	99	29

### Stage 3: Evaluation

#### Allied HCPs (Students to Specialists)

**Figure 3:**
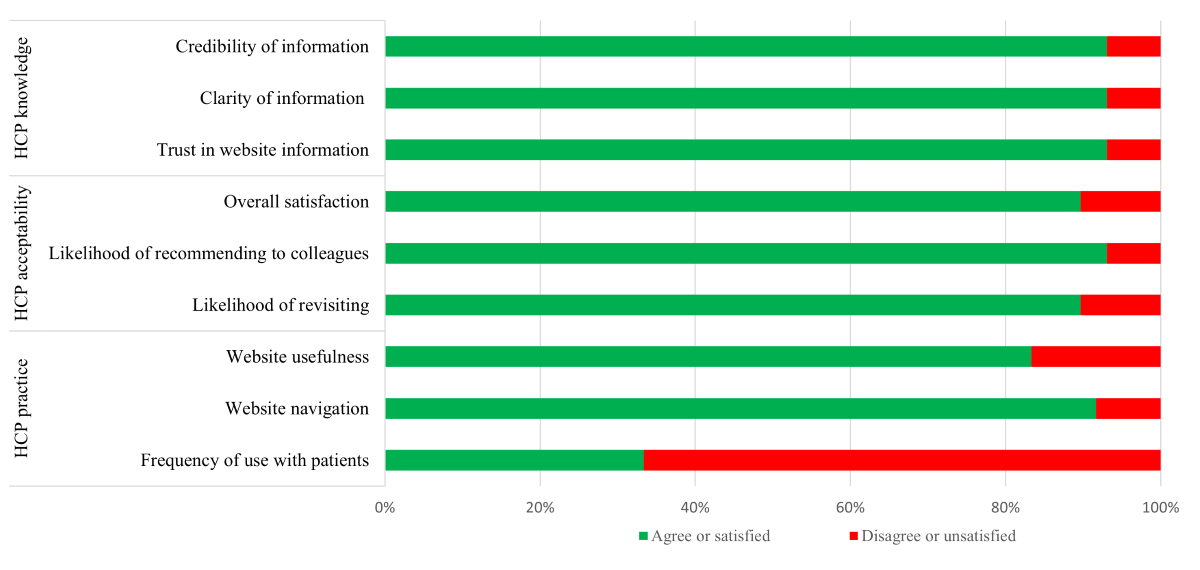
Health care professionals’ (HCP) opinions about MyPainHub. Opinions on knowledge and acceptability were positive, but this contrasted to how often HCPs used MyPainHub with patients.

Of the 102 allied HCPs invited, 29 (28%) HCPs completed the evaluation questionnaire. Of these, 93% (27/29) responded that MyPainHub had the potential to change knowledge as the information contained on the website was credible, trustworthy, clearly presented and easy to navigate. In terms of acceptability of the website, 93% (27/29) of HCP participants were satisfied with the website, were likely to revisit, and were likely to refer other colleagues to the site. However, less than 35% (10/29) of HCPs reported using the website with their patients. Suggestions for improvement included considering changing some of the information to enhance understanding for people with low health literacy and to include other musculoskeletal conditions (HCPs’ opinions are shown in Figure 3).

From the focus group (6 HCP participants), four key themes emerged: (1) MyPainHub has utility as a web-based resource; (2) MyPainHub contains credible information, which reinforces best practice; (3) challenges in implementation; and (4) potential opportunities for enhancement.

##### Theme 1: MyPainHub Has Utility as a Web-Based Resource

HCP participants reported MyPainHub contained relevant information, was easy to navigate and improved professional confidence. HCPs supported the relevance and breadth of topics on the site and its utility in enhancing quality of care, “especially for junior staff or those with lesser experience.” Despite challenges with implementation, participants had felt that it was useful for “encouraging patient engagement with HCPs,” fostering an educational continuum, and “...builds on education and use of resources between sessions based on patient learning preferences.” The “easy to navigate” resource had increased professional confidence as it “could help through processes, guide treatment and reduce fears when unsure or confused.” This theme highlighted the website’s potential in bridging knowledge gaps and facilitating a more informed patient-provider interaction.

##### Theme 2: MyPainHub Contains Credible Information, Which Reinforces Best Practice

The HCPs reported information on MyPainHub was credible and aligned with best practice, particularly because of the inclusion of video content and articles from expert clinicians and researchers. Comments regarding the information on MyPainHub “reinforced stuff previously learnt at university” and “reinforced what we already say (to patients) in a different format.” Some remarked that the resource helped their own learning around best practice which in turn would influence the education they provided to their patients: “I don't have too much outpatient experience nor am I very confident. So I really like the clinician end of it...using that to understand things for myself and then being able to explain that to someone.”

##### Theme 3: Challenges With Implementation

HCP participants reported implementation of MyPainHub was harder than expected and identified several challenges including internet availability in the clinic, time constraints, and a lack of familiarity with using web-based resources to support patient interactions. Acknowledging “change in practice is difficult to achieve in busy clinical settings,” participants identified that “more training and preparation to support new workflows is needed.” HCPs perceived those patients “may expect a more hands-on approach” which made them “hesitant” to adopt MyPainHub within sessions. As some HCPs were “not accustomed to using online resources” to aid their management of patients, they acknowledged they “often forgot about MyPainHub as an option,” particularly in the context of “competing demands and heavy clinical workloads.”

##### Theme 4: Potential Opportunities for Enhancement

Opportunities for improvement were noted, including the simplification of how information was presented, reduction of hyperlinks, and expansion of the number of conditions included on the site. HCPs voiced the need for the key messages and information to be “reduced” and “summarized” so that users would not have to “scroll as much.” Some participants suggested that “more downloadable resources may be more helpful” and proposed more “ready-made printouts and resources of information.”

#### People With Musculoskeletal Conditions

Eleven people with musculoskeletal conditions in the tertiary setting (WSLHD) completed the evaluation questionnaire. All responded that MyPainHub had the potential to change knowledge as the information contained on the website was credible, trustworthy and clearly presented. In terms of acceptability, all participants were satisfied with MyPainHub, likely to revisit and recommend the website to others. In addition, all participants indicated that the web-based resources helped them understand the best ways to manage their condition, the website was easy to navigate, and that MyPainHub would be a useful tool for other people with musculoskeletal conditions.

Four key themes emerged from the focus group (n=6 participants) from participants at WSLHD and these aligned with the findings from the questionnaire: (1) MyPainHub adds to the understanding of the condition, (2) MyPainHub is credible as it aligns with HCP advice and information, (3) MyPainHub supports self-management, and (4) potential areas for improvement.

##### Theme 1: MyPainHub Adds to the Understanding of the Condition

Participants reported information on MyPainHub added to the understanding of their musculoskeletal condition. It was reported that the website offered clear explanations that were “definitely helpful,” “adding knowledge and understanding,” and “changing their perspectives on their condition.” This aspect was further supported by the ability to “revisit information” via the “links” as needed, allowing for engagement with the content and facilitating ongoing learning.

##### Theme 2: MyPainHub Is Credible as it Aligns With HCPs’ Advice and Information

Participants reported information on MyPainHub as credible, particularly when it aligned with their HCP’s advice and information. This alignment reassured patients and “built trust” in both the website content and the advice provided by their HCPs. Trust was further reinforced when the website was recommended by their HCP or “experts.”

##### Theme 3: MyPainHub Has the Potential to Support Self-Management

Participants reported the website increased their confidence in managing their condition. This was achieved by supporting “self-help and lifestyle changes” and “promoting exercise, and providing a holistic approach to physical activity and mental health.” The ability to “support more activity and distract from negative thoughts” was reported, underlining the website’s role in supporting both physical and psychological aspects of self-efficacy.

##### Theme 4: Potential Areas for Improvement

Participants identified areas for improvement. This included the need for more specific information tailored to “work or employment challenges,” “detailed do’s and don’ts for condition management,” “preventative measures,” “pain relief options,” and “information about surgery and when it may be needed.” “Regular updates” and the creation of “shared chat groups” were also suggested as potential areas for improvement.

## Discussion

### Principal Findings

This paper describes the process undertaken to develop, implement and evaluate a website designed to support patients with, and HCPs who manage, musculoskeletal conditions, specifically LBP, neck pain, WAD, and knee osteoarthritis. The findings suggest that following a research translation framework and incorporating extensive and iterative consultation with multiple key stakeholders resulted in MyPainHub being acceptable, credible, and easy to navigate with participants reporting they are likely to revisit and recommend the website to others. Embedding prognostic tools (the SF-ÖMPSQ and the Keele STarT MSK tool) allowed information to be tailored to a person’s risk of poor outcome which may help both patients and HCPs better adopt guideline-based management. However, training for HCPs that includes more specific strategies on how to incorporate MyPainHub into clinical practice would be beneficial for more successful implementation.

A key component of the development of MyPainHub was the extensive involvement of a wide range of key stakeholders with different levels of experience and from different health care settings over a 4-year period. The initial development phase focused on gathering perspectives from people with musculoskeletal conditions and HCPs from primary care settings, researchers, key opinion leaders, professional associations, consumer groups and website developers. The latter development phase involved seeking perspectives of HCPs and patients from tertiary care settings, allied professionals from occupational settings and from allied health students. This broad stakeholder engagement reflects contemporary user-centered design and was undertaken to facilitate future wider implementation of MyPainHub across a range of settings and populations [61]. Whilst active implementation strategies (eg, interactive education and workshops) were generally more effective than passive strategies (eg, phone calls and emails), implementing MyPainHub in a unit of study was not successful amongst allied health students. In our study, students were introduced to MyPainHub during a lecture and were then responsible for designing a treatment program using MyPainHub but no specific tutorial time was allocated for this task. A previous study [43] that had implemented an innovative, guideline-based website, My Whiplash Navigator*,* found student HCPs had a higher uptake of the website than primary HCPs but the students were allocated time during both a lecture and a tutorial to using the resource which may explain the difference in the level of engagement.

The main purpose of this project was to develop a resource that would support guideline-based care for patients at low and high risk of poor outcome. Tailoring information to a person’s risk of poor outcome afforded the opportunity to provide different information on likely recovery pathways and interventions that may be required. In this way, MyPainHub reinforces key guideline-based messages, that people at low risk should do well with minimal care but that people at high risk may require more comprehensive assessment of multiple domains and a multidisciplinary approach to optimize health outcomes. Greater adoption and wider implementation of MyPainHub may improve the efficiency of health service delivery by helping to educate people about musculoskeletal conditions and influence care decisions but requires further research.

Despite the opinions of stakeholders that MyPainHub is acceptable, credible, and easy to navigate, few used the tools with patients, and additional training that focuses on how best to integrate the website into clinical practice in different health care settings is likely to be required for greater adoption and usage. Active engagement strategies were more effective than passive strategies and taking a multifaceted approach, for example embedding MyPainHub in existing educational opportunities (eg, workshops, curriculum, and training programs) and using key opinion leaders to reinforce the credibility of information, is likely to be important in future implementation [62,63]. The poor response rate and level of engagement of students could be overcome by integrating MyPainHub as a supplementary resource into the physiotherapy program curriculum and ensuring the information provided aligns with the learning objectives of the specific unit of study with which it is associated. Effective incorporation of MyPainHub for students may be achieved through thoughtful planning and implementation, integrating into specific lesson plans including activities such as group discussions followed by monitoring of student engagement via Learning Management Systems or linked to assessable content [64].

As with any web-based resource, updates to MyPainHub are ongoing with the maintenance of the website being the responsibility of the research team and the digital agency. Addition of information about other conditions (eg, hip pain) is underway and incorporating additional features such as more dynamic elements (eg, carousels and expandable sections) and gamification to increase engagement [65] are likely to be important features of future updates. Limitations of this study include that we did not control the time between participants viewing the website and asking for their feedback. Despite efforts to engage general practitioners and other medical professionals, the majority of those involved in the development of MyPainHub were allied HCPs. Further work is needed to determine whether MyPainHub, or specific sections of MyPainHub, would be useful for general practitioners. Further work is also required to implement MyPainHub in other primary (eg, community-based care) and tertiary care settings.

### Conclusion

This study details the development, implementation, and evaluation of MyPainHub, a web-based resource designed to support guideline-based care for musculoskeletal conditions. Stakeholder engagement throughout the process contributed to its acceptability, credibility, and ease of use. While MyPainHub effectively tailors information based on prognostic tools to support both patients and health care providers, further efforts are needed to facilitate its integration into clinical practice. Training for health care professionals, particularly in how to apply MyPainHub in diverse settings, is likely essential for greater adoption.

## Appendix

Multimedia Appendix 1 Healthcare professional survey.

## Abbreviations

HCP: health care professional
LBP: low back pain
MSK: muscoluskeletal
PACE: PAthway of CarE
RE-AIM: Reach, Effectiveness, Adoption, Implementation, and Maintenance
SF-ÖMPSQ: Short Form Örebro Musculoskeletal Pain Screening Questionnaire
WAD: whiplash associated disorders
WSLHD: Western Sydney Local Health District

## Data Availability

The datasets generated or analyzed during this study are available from the corresponding author on reasonable request.
