# Supplementary figures and images for: Development and Implementation of MyPainHub, a Web-Based Resource for People With Musculoskeletal Conditions and Their Health Care Professionals: Mixed Methods Study
Source: JMIR Form Res. 2025 Feb 24;9:e63780. doi: 10.2196/63780 (PMC11894348; doi:10.2196/63780)

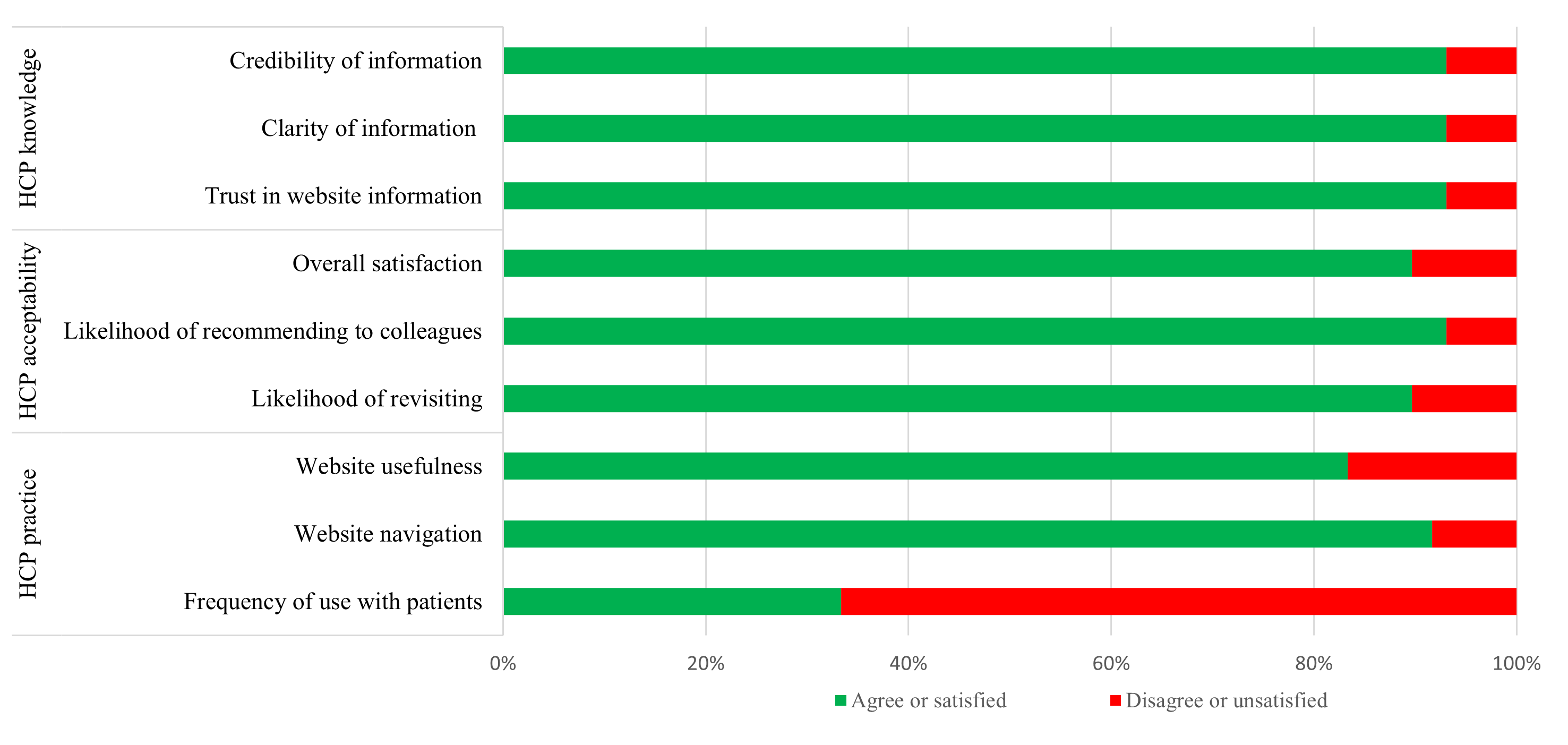

Supplement: Multimedia Appendix 1 [file formative_v9i1e63780_app1.png]
